# Rational thinking as a mediator of the relationship between mindfulness and dental anxiety

**DOI:** 10.1038/s41598-023-28879-4

**Published:** 2023-02-22

**Authors:** Jiaxuan Yao, Richard Carciofo, Li Pan

**Affiliations:** 1 Department of Social Statistics, The University of Manchester, HBS Building, Oxford Road, Manchester, M13 9PL UK; 2 International Business School Suzhou, Xi’an Jiaotong-Liverpool University, No. 8 Chongwen Road, Suzhou, China; 3 School of Psychology and Clinical Language Sciences, University of Reading, Reading, UK

**Keywords:** Psychology, Dentistry

## Abstract

Dental anxiety causes people to postpone or refuse to go to the dentist, which further negatively impacts their quality of life and public health. Previous research has shown that mindfulness is inversely related to anxiety. However, little is known about the relationship between mindfulness and dental anxiety. The current research aimed to explore the relationship between mindfulness and dental anxiety and investigate the mediating role of rational thinking. Two studies were conducted. In study one, 206 Chinese participants completed questionnaires measuring trait mindfulness and dental anxiety (state, responding to a dental treatment scenario). In study two, 394 participants completed questionnaires measuring trait mindfulness, dental anxiety (trait), and rational thinking. The results for both studies showed that mindfulness is negatively correlated with dental anxiety. In study 1, each facet of mindfulness except Non-judging was negatively correlated with dental anxiety with Acting with Awareness having the strongest correlation, while in study 2, only Acting with Awareness was significantly negatively correlated with dental anxiety. Furthermore, rational thinking mediated the effect of mindfulness on dental anxiety. In conclusion, mindfulness is negatively related to both state and trait dental anxiety, and rational thinking mediates the relationship between mindfulness and dental anxiety. Implications of these findings are discussed.

## Introduction

### Dental anxiety

Dental anxiety is anxiety related to dental treatment, such as fear of the unknown and pain^1^. It is characterized by anxious thoughts about dentistry and fear reactions in the dental treatment, and the two terms, dental fear and dental anxiety are often used in the literature interchangeably^2^. Dental anxiety is prevalent among children and adults, people from eastern countries^3^ and western countries^4^. Examining dental anxiety is important as dental anxiety causes people to postpone or refuse to go to the dentist^5^, which further negatively impacts their quality of life^6^ and public health^7^.

Negative experiences inherent in dental service is one of the major causes of dental fear^8^. Negative sensory stimuli such as noise from drills, smell of eugenol and vibration sessions often trigger dental anxiety^9–11^. Among various traumatic treatments, such as teeth grinding, fillings, and drilling, tooth extraction is the one that most likely makes patients feel anxious^12^.

However, negative experiences do not necessarily cause dental anxiety, which may be attributed to individual differences. Roy and Dempster^1^ found that future dental patients who have not had any unpleasant dental treatment in the past reported higher levels of dental anxiety than current patients. Similarly, among patients who experienced negative dental experiences, there were those who did not suffer from dental phobia^13^. These findings indicate a complex relationship between having had negative experiences and having dental anxiety, which may be influenced by various individual differences which moderate or mediate this relationship. For example, previous research found that women, younger individuals, and people who do not exercise regularly are at greater risk for dental anxiety^5^.

Dental anxiety is a complex phenomenon with somatic, psychological, and social dimensions^2^. Previous literature on relieving dental fear and anxiety has mainly focused on two approaches: medical and psychological. Medical interventions comprise both established measures like sedation and general anesthetic^14^ that are widely used, and measures that are under ongoing study, such as auricular acupuncture^15^ and Respiratory Rate Biofeedback Device^16^. Psychological interventions include Cognitive Behavior Therapy^9,17^, hypnosis^18^, desensitization therapy^19^, music^20^, and aromatherapy^21^.

### Mindfulness and dental anxiety

Mindfulness was used in western psychotherapy for the first time in the 1980s, primarily for patients who suffered from various organic diseases, to relieve their stress and pain^22^. Jon Kabat-Zinn^23^ introduced mindfulness training into the Western medical system. As he defined, mindfulness is the awareness that emerges through paying attention in a special way: on purpose, in the present moment, and nonjudgmentally^23^. Baer et al.^24^ described mindfulness as habitual awareness of thoughts and feelings at the moment and found that mindfulness consists of five factors/facets: observing cognitive and physical experiences; the ability to verbally describe one’s experiences; acting with awareness; non-judging of inner experiences; and non-reactivity to inner experience (not becoming absorbed in thoughts or feelings).

Much research has supported the positive effect of mindfulness on anxiety. Higher levels of dispositional mindfulness have been consistently found to be correlated with being less anxious^25^. Mindfulness-based Intervention (MBI) has been found to be effective in improving patients’ level of trait mindfulness, including mindfulness assessed with self-report measures^26–28^, which may further help people with various anxiety-related disorders^29–31^ Various research has also demonstrated that mindfulness is beneficial to specific types of anxiety, such as social anxiety^32^ and math anxiety^33^^.^

It is worthwhile to further investigate the potential benefits that mindfulness has to the medical field. When mindfulness training was first introduced to the Western medical system, those who participated were found to have fewer hospital visits and medical problems^34^. Research on cancer patients^35,36^ shows that increasing one’s level of mindfulness can reduce anxiety about future medical problems, and specifically anxiety about a medical treatment. In dental treatment, Whitaker^37^ designed a short mindfulness-based technique that could manage dental fear. Li et al.^38^ found that a Mindfulness-based Stress Reduction (MBSR) program had better long-term effects on reducing dental anxiety compared with music therapy in patients for implant denture. It has also been found that mindfulness training with children can reduce their dental anxiety compared with a control group^39^. However, Vaught’s^40^ study on college students in the USA, using the unidimensional measure Mindful Attention Awareness Scale (MAAS)^34^ to assess mindfulness, found that mindfulness was not a significant predictor of dental anxiety, while Tellez et al.’s^41^ study with American dental patients found that, of the five facets of mindfulness, only the Acting with Awareness and Non-judging facets were negatively associated with dental anxiety. Thus, in summary, previous research has shown some support for the negative association between mindfulness and dental anxiety, however previous studies only included trait measures of dental anxiety and the results have been somewhat inconsistent and limited to specific samples. Furthermore, previous research has not investigated possible mechanisms that may be involved in the relationship between mindfulness and dental anxiety.

### The role of rational thinking

Previous research investigating the negative association between mindfulness and anxiety has been mainly focused on mechanisms related with affect. Less experiential avoidance^42^, less affective reactivity^43^, higher emotional intelligence^44^, and higher emotional regulation^45,46^ have been found to account for the inverse relation between mindfulness and anxiety. Emotional responses are typical in the experiential system, however there is another parallel, interactive system for processing information, the rational system, as proposed by Cognitive-experiential self theory (CEST)^47^. Individuals differ in the degree to which they characteristically operate in one mode or the other^48^. When individuals employ the rational system, they are likely to override emotional responses that are typical in the experiential system, which is achieved through cognitive decoupling, the ability to distinguish supposition from beliefs and re-represent emotional experience, allowing individuals to consciously determine the best course of action^48^. It is worthwhile to investigate the role that rational thinking may have in the relationship between mindfulness and anxiety. Mindfulness enables individuals to realize that thoughts and feelings are simply transient mental events that continuously rise and fall from conscious awareness^49^. This realization leads to the creation of mental distance from present thoughts and emotions (cognitive decoupling), which results in individuals being able to respond to the event from a conscious level rather than an unconscious level^50^. Previous research on the relationship between mindfulness and rational thinking is very limited, but provides support for the positive association between mindfulness and rational thinking. Farrar and Tapper^51^ found a significant positive correlation between mindfulness and rational thinking (r = 0.56). Further analysis showed that all mindfulness subscales (Observing, Describing, Non-reacting, and Acting with Awareness), except for the subscale of Non-judging, were positive predictors of rational thinking. Kirk et al.^52^ also found that mindfulness meditation practitioners are more rational and better at regulating emotions compared with control groups when making decisions for an ultimatum game.

To our best knowledge, no previous research has examined how individual differences in the tendency to rely on rational thinking may influence anxiety or dental anxiety. However, some related research topics suggest that people with a higher level of rational thinking are more immune to anxiety problems. Rational Emotive Behavioral Therapy (REBT)^53^, a cognitive behavioral approach that targets at weakening irrational beliefs and strengthening rational beliefs, is particularly effective with anxiety-related issues^54^. Previous findings have highlighted the link between rational beliefs and less anxiety^55^, stronger ability to deal with the stress of negative life events^56^, and more tolerance in stressful situations^57^. Based on the preceding rationales and available literature showing that mindfulness contributes to the rational thinking mode and that the rational thinking mode is potentially negatively related with anxiety, it may be proposed that rational thinking mediates the relationship between mindfulness and dental anxiety.

### The present study

The first aim of the study was to test the relationship between mindfulness facets and both state and trait dental anxiety, in an adult student population. Secondly, we assume mindfulness will indirectly predict lower levels of dental anxiety through rational thinking. To the best of our knowledge, no previous research has investigated the effect of mindfulness on dental anxiety through rational thinking. The hypothesis described above was tested in two studies. In Study 1, participants were recruited through an online subject pool platform in China and state anxiety responding to a dental treatment was measured to test the associations of mindfulness facets with dental anxiety. Study 2 was conducted among students in a Chinese university; trait dental anxiety and rational thinking were measured to further verify the association between mindfulness and dental anxiety, and the role that rational thinking may have in this relationship. Measuring dental anxiety with both state and trait scales will increase the validity of our results. For both studies, The Five-Facet Mindfulness Questionnaire (FFMQ)^24^ was used to measure mindfulness.

## Study 1 

### Overview

This study measured mindfulness using the FFMQ scale and dental anxiety using the STAI-State scale to measure dental anxiety in response to a dental treatment. The relationship between mindfulness, each of its five factors, and dental anxiety were examined.

### Participants and procedures

#### Research sample recruitment

Data collection was conducted through an online questionnaire platform called Questionnaire-Star (https://www.wjx.cn). Participants were randomly recruited from the subject pool of Questionnaire-Star. The eligibility criteria for this study required the sample to be students from Chinese universities, with a gender ratio of 1:1 for male and female, and the participants should be at least 18 years old. Cohen’s^58^ guidelines suggest that small, medium, and large effect sizes may be indicated by correlation coefficients of 0.10, 0.30, and 0.50, respectively, and medium effect sizes may be established with 80% power at the 5% significance level with a sample size of N = 85, while for small effect sizes N = 783 is suggested. The target number of participants was set at 200. This was adequate to establish small to moderate correlations (r = 0.20) at the 5% significance level with 80% power^59^.

The questionnaire was released on 26 March 2020 and the target sample size was reached on 30 March, with 206 participants, from 28 provinces in China, having completed the self-report online survey questionnaire with their own devices, such as mobile phones, pads, and laptops. Each recruited participant was paid. See the Supplementary Materials for the full sample description.

### Ethical approval 

This study was approved by the Xi’an Jiaotong-Liverpool University (XJTLU) Research Ethics Committee (Approval number: 10-04-13). Participants read the information sheet and consent form, which included contact information for the researchers. Informed consent was obtained from all participants. Participation was voluntary and could be withdrawn at any time. All methods were performed in accordance with the regulations and guidelines.

### Measures

#### Mindfulness

The FFMQ^24^, a 39-item scale, was used to assess the level of mindfulness. We used the Chinese version of the FFMQ translated by Deng et al.^60^. The FFMQ includes five factors (Observing, Describing, Acting with Awareness, Non-judging, and Non-reacting). Example items are: Observing, *When I’m walking, I deliberately notice the sensations of my body moving*; Describing, *I’m good at finding words to describe my feelings*; Acting with Awareness, *When I do things, my mind wanders off and I’m easily distracted* (reverse-scored); Non-judging, *I tell myself I shouldn’t be feeling the way I’m feeling* (reverse-scored); Non-reacting, *I watch my feelings without getting lost in them*.

Items are rated on a five-point Likert-type scale, ranging from 1 (never or very rarely true) to 5 (very often or always true). The FFMQ has been used as an outcome measure to assess changes in mindfulness-based intervention studies^27,28,61^. All five factors have demonstrated at least adequate internal consistency in past research, ranging from 0.73 to 0.91^62^ and from 0.448 to 0.843 for the Chinese scales^16^. Internal consistency was excellent in the present sample for the total scale score (Cronbach's α = 0.797). Cronbach’s α in the current sample was 0.693 for Observing (eight items), 0.846 for Describing (eight items), 0.816 for Acting with Awareness (eight items), 0.601 for Non-judging (eight items), and 0.522 for Non-reacting (seven items). The low alpha for the Non-reacting subscale is consistent with Deng et al.’s^60^ research.

#### Dental anxiety

The most widely used tool for evaluating the state of anxiety in dental patients is the State Trait Anxiety Inventory-State with 20 items^63^. We used the short version of this scale to examine dental anxiety, which according to previous research is reliable (Cronbach’s α = 0.83) and positively correlates (r = 0.89) with the 20-item scale^64^. This study used the Chinese translation of state anxiety by He et al.^65^. The short STAI-State scale includes 7 items which include two dimensions: anxiety (Example: I feel worried) and non-anxiety (Example: I feel happy). It is a four-point Likert scale, with 1 'not at all' to 4 'very much so' ratings. The scores of non-anxiety items were reversed. Individuals with higher overall scores have higher levels of dental anxiety.

This scale was administered after participants read a dental service consumption scenario to measure participants’ state dental anxiety. With a displayed advertising poster, the respondents were asked to imagine that they went to consult about an orthodontic service at a service desk of a private dental hospital located in the hall of a subway station (a common marketing practice of private dental hospitals in China). After initial examination of their oral condition, the participants were told that the consultant introduced Invisalign clear aligners that are more comfortable than braces, but accordingly to their teeth condition, he/she is highly likely to have one of their teeth extracted. We designed this scenario based on a pilot study which found that for patients who are interested in orthodontic service, extracting teeth is what could arouse their dental anxiety. In the pilot study, 40 participants were randomized into two groups. Group One read an orthodontic service introduction that included information about extracting teeth, while Group Two's introduction did not contain any information about extracting teeth. After reading the introduction, both groups reported their dental anxiety level measured by the STAI-State scale. Results showed that the dental anxiety of participants in Group One (mean = 3.74, SD = 1.16) was significantly higher than that of participants in Group Two (mean = 2.407, SD = 0.925; t = 4.010, p < 0.001).

### Results

#### Participant demographics and correlation analyses

Among the 206 participants, the average age was 20.66 (mean = 20.66, SD = 1.651, range = 18 to 25). 108 of them were male (age: mean = 20.80, SD = 1.723) and 98 were female (age: mean = 20.50, SD = 1.561). Participants who scored higher on mindfulness were older (r = 0.15, p < 0.05), but gender was not correlated with mindfulness. The full details of descriptive statistics and correlations are provided in Table 1. As expected, we found that mindfulness (FFMQ total score) had a medium negative correlation with dental anxiety (r = − 0.32, p < 0.01). Of the FFMQ facets, dental anxiety showed significant small to medium negative correlations with Observing, Describing, Acting with Awareness and Non-reacting, but Non-judging was not significantly correlated.

**Table 1:** Descriptive statistics and correlations between primary variables (study 1).

	M	SD	1	2	3	4	5	6	7	8
1. Dental anxiety	1.95	0.58	1							
2. Observing	3.28	0.67	− 0.20**	1						
3. Describing	3.09	0.75	− 0.18**	0.31**	1					
4. Acting with awareness	3.01	0.78	− 0.22**	0.08	0.43**	1				
5. Non-judging	2.71	0.54	− 0.13	− 0.09	0.11	0.28**	1			
6. Non-reacting	2.99	0.56	− 0.20*	0.25**	0.08	− 0.05	− 0.17*	1		
7. Mindfulness	3.01	0.38	− 0.32**	0.55**	0.76**	− 0.70**	0.39**	0.30**	1	
8. Age	20.66	1.65	− 0.01	0.02	0.15*	0.16*	0.05	0.01	0.15*	1
9. Gender (male = 0)	0.48	0.50	0.13	0.03	0.04	0.01	0.14*	− 0.04	0.07	− 0.09

*p < 0.05, **p < 0.01, n = 206.

Significant values are in bold.

#### Regression analyses

A forced entry multiple regression (Table 2, Model 1) showed that FFMQ total score, and gender, were significant predictors of dental anxiety. A second regression (Table 2, Model 2) utilizing the five factors showed the correlation between the five subscales of the FFMQ and dental anxiety was moderate to strong (Multiple R = 0.38, p < 0.01) with the subscales (plus age and gender) accounting for 11% of the variance in dental anxiety (adjusted R^2^). The data analyses indicated that multicollinearity was not problematic (all Variance Inflation Factor values were < 1.4). Three of the subscales negatively predicted dental anxiety: Observing, Non-judging, and Non-reacting, while Acting with Awareness’s negative prediction was approaching significance (p = 0.052) and Describing’s negative prediction was not significant.

**Table 2:** Study 1 regression results.

	Model 1	Model 2
	DV = dental anxiety	DV = dental anxiety
Mindfulness	− 0.34**	–
Observing	–	− 0.15*
Describing	–	− 0.06
Acting with awareness	–	− 0.15
Non-judging	–	− 0.15*
Non-reacting	–	− 0.17*
Age	0.06	0.05
Gender	0.16*	0.16*
R^2^ adjusted	0.114	0.110
F	9.834	4.625

*p < 0.05, **p < 0.01.

### Discussion

In study 1, results showed that individuals with higher levels of mindfulness have lower dental anxiety, after controlling age and gender. Even though the correlation analysis showed that all five facets of mindfulness except Non-judging were significantly negatively correlated with dental anxiety, a forced entry multiple regression showed that Non-judging became a significant negative predictor of dental anxiety, as were Observing and Nonreactivity, while Acting with Awareness’s negative effect was approaching significance and Describing’s negative effect was nonsignificant.

## Study 2

### Overview

Study 2 measured trait dental anxiety using the Modified Dental Anxiety Scale (MDAS) and mindfulness using the FFMQ scale. We expected people with higher scores on mindfulness to report lower levels of dental anxiety. The relationship between each facet of mindfulness and dental anxiety was also explored. We further measured individual differences in rational thinking to examine whether rational thinking mediates the relationship between mindfulness and dental anxiety. We also examined whether rational thinking mediates the relationship between each facet of mindfulness and dental anxiety.

### Participants and procedure

Beginning on 19 March 2021, participants were recruited from a course for undergraduates at Xi’an Jiaotong-Liverpool University, which is an international, English-medium university located in Suzhou, Jiangsu Province, China. The questionnaire was presented bilingually, in both English and Chinese and released online through the Questionnaire-Star platform. Students who took the survey were compensated 5 course credits. A replacement task was provided if they did not want to do the survey task. The researchers released the questionnaire on 21 March and completed data collection on 29 March; 394 students from over 4 countries finished the survey task within 9 days in exchange for course credit. See the Supplementary Materials for the full sample description. The Ethical approval statement was the same as for Study 1.

### Measures

The self-report questionnaires were composed of the FFMQ scale for mindfulness, the MDAS scale for dental anxiety and the Rational Experiential Inventory scale (REI) for rational thinking. Internal consistency for mindfulness (FFMQ total score, Cronbach's α = 0.768) was excellent for this sample as well. Cronbach’s α in the current sample was 0.760 for Observing, 0.858 for Describing, 0.829 for Acting with Awareness, 0.721 for Nonjudging, and 0.544 for Nonreactivity.

#### Dental anxiety

Participants’ dental anxiety was measured by the Modified Dental Anxiety Scale (MDAS) developed by Humphris et al.^8^, which is adapted from Corah’s dental scale^66^. This scale is widely used to measure an individual's trait level of dental anxiety. The Chinese version of the MDAS translated by Ng et al.^67^ was used for our questionnaire. An example item is: *If you went to your dentist for treatment tomorrow, how would you feel?* The scale includes five questions, each with five category rating scales ranging from “no anxiety” to “extreme anxiety”. Internal consistency of the MDAS was excellent in the present sample (Cronbach's α = 0.871).

#### Rational thinking

Participants’ rational thinking was measured by the Rational Experiential Inventory scale (REI) developed by Epstein et al.^47^. Rational thinking style comprised 19 items. An example item is: *I would prefer a task that is intellectual, difficult, and important to one that is somewhat important but does not require much thought*. For this study, we used the Chinese version of the REI translated by Wen^68^. Reliability for the rational thinking subscale was excellent (Cronbach’s α 0.874) in our sample.

### Results

#### Participant demographics and correlation analyses

The final sample included 394 respondents. The average age was 18.62 (SD = 0.96), ranging from 16 to 26. 170 participants were male (age: mean = 18.77, SD = 1.172) and 224 were female (age: mean = 18.50, SD = 0.734). Nationality was as follows: 91.9% Chinese, 3.6% Korean, 2.5% Indonesia, 2.0% Other. Mindfulness positively correlated with age with a small/medium coefficient (r = 0.17, p < 0.01), and participants who scored higher on dental anxiety were more likely to be female (r = 0.13, p < 0.01). The full details of demographic variables are provided in the Supplementary Materials.

Table 3 shows descriptive statistics and the correlations for the research variables. As expected, we found that mindfulness had a significant negative correlation with dental anxiety, with a small coefficient (r = − 0.14, p < 0.01), and a significant, medium/strong positive correlation with rational thinking (r = 0.43, p < 0.01). Additionally, dental anxiety had a small/medium negative correlation with rational thinking (r = − 0.18, p < 0.01).

**Table 3:** Descriptive statistics and Correlations between primary variables (study 2).

	M	SD	1	2	3	4	5	6	7	8	9
1. Dental anxiety	3.07	0.95									
2. Rational thinking	3.32	0.54	− 0.18**								
3. Observing	3.42	0.62	− 0.03	0.26**							
4. Describing	3.19	0.66	− 0.09	0.30**	0.26**						
5. Acting with awareness	3.30	0.63	− 0.11*	0.33**	− 0.10	0.39**					
6. Non-judging	2.76	0.54	− 0.07	0.07	− 0.43**	− 0.003	0.34**				
7. Non-reacting	2.99	0.48	− 0.05	0.05	0.43**	− 0.14**	− 0.16**	− 0.30**			
8. Mindfulness	3.13	0.30	− 0.14**	0.43**	0.46**	0.77**	0.64**	0.25**	0.35**		
9. Age	18.62	0.96	− 0.05	0.001	0.12*	0.17*	0.09	0.02	0.08	0.17**	
10. Gender (male = 0)	0.57	0.50	0.13**	− 0.04	− 0.01	− 0.01	0.05	0.09	− 0.07	0.03	− 0.14**

*p < 0.05, **p < 0.01, N = 394.

Significant values are in bold.

For the five factors of mindfulness, Rational thinking had significant medium positive correlations with the Observing, Describing, and Acting with Awareness facets of mindfulness, and dental anxiety only had a significant small negative correlation with the Acting with Awareness factor.

#### Regression analyses

When controlling for age and gender, mindfulness (FFMQ total score) was a significant predictor of rational thinking (Table 4, model 1). When testing the five FFMQ facets, all were significant predictors of rational thinking except for Non-reactivity (Table 4, model 3). Age and gender were not significant in either model.

**Table 4:** Study 2 regression results.

	DV = rational thinking	DV = dental anxiety	DV = dental anxiety	DV = rational thinking	DV = dental anxiety	DV = dental anxiety
	Model 1	Model 2a	Model 2b	Model 3	Model 4a	Model 4b
Mindfulness	0.443**	− 0.139**	− 0.078	–	–	–
Observing	–	–	–	0.327**	− 0.035	0.013
Describing	–	–	–	0.118*	− 0.030	− 0.013
Acting with awareness	–	–	–	0.289**	− 0.095	− 0.053
Non-judging	–	–	–	0.106*	− 0.076	− 0.061
Non-reacting	–	–	–	− 0.027	− 0.057	− 0.061
Rational thinking	–	–	− 0.137*	–	–	− 0.146*
Age	− 0.082	− 0.010	− 0.021	− 0.087	− 0.010	− 0.023
Gender	− 0.059	0.135**	0.127*	− 0.070	0.138**	0.128*
R^2^ adjusted	0.185	0.030	0.043	0.212	0.023	0.038
R^2^ changed	0.191	0.037	0.053	0.26	0.041	0.057
F	30.704	5.031	5.389	16.137	2.350	2.923

*p < 0.05, **p < 0.01.

Significant predictors of dental anxiety were tested with hierarchical linear regression (Table 4). Firstly, in model 2a, mindfulness (FFMQ total score), age, and gender were entered as predictors; then in model 2b, rational thinking was entered as an additional predictor. For model 2a, the significant predictors were mindfulness and gender. Model 2b produced a significant change (F = 5.389, p < 0.001; change in R^2^ = 0.053), and in this model the significant predictors were rational thinking and gender; mindfulness was no longer significant.

Model 4a, included all five FFMQ subscales, age, and gender, but none of the FFMQ facets were significant predictors of dental anxiety (only gender had a significant effect). Model 4b also included rational thinking, which produced a significant change in the model (F = 2.923, p = 0.004; change in R^2^ = 0.057), and in this model the significant predictors were rational thinking and gender. The data analyses indicated that multicollinearity was not problematic in either model 4a or 4b (all Variance Inflation Factor values were < 1.7).

Separate regression models for each facet individually, also including age and gender (see Supplementary Materials), showed that only the Acting with Awareness facet was a significant predictor of dental anxiety. When rational thinking was added as an additional predictor, Acting with Awareness was no longer significant. Gender was a significant predictor in all models.

#### Mediation analyses

As the hierarchical regression showed that mindfulness (FFMQ total) was no longer significant after rational thinking was included in the model (Table 4, model 2b), it was tested whether rational thinking mediates between mindfulness (FFMQ total), and dental anxiety.

Mediation was formally tested using the SPSS PROCESS macro with 5000 resamples^68^. Table 5 shows the unstandardized results. The indirect effect of mindfulness on dental anxiety through rational thinking was supported (95% CI = − 0.3640 to − 0.0263). The negative relation between mindfulness and dental anxiety was mediated by rational thinking. Table 5 shows that this mediating (indirect) effect accounted for 43.81% of the total effect.

**Table 5:** Mediation results: the indirect effect of mindfulness on dental anxiety through rational thinking.

	Effect	SE	LLCI	ULCI	Effect ratio (%)
Total effect	− 0.4355	0.1585	− 0.7471	− 0.1239	
Direct effect	− 0.2448	0.1749	− 0.5887	0.0991	56.21
Indirect effect	− 0.1908	0.0862	− 0.3640	− 0.0263	43.81

Likewise, as the Acting with Awareness facet was no longer a significant predictor after rational thinking had been included in the regression model (Supplementary Materials), it was tested whether rational thinking mediates between this facet and dental anxiety. However, the indirect effect was not significant (95% CI = − 0.0758 to 0.0227; see Supplementary Materials).

Given that indirect effects may exist between the predictor and dependent variable even in the absence of a significant association (total effect)^69^, further exploratory bootstrapping analysis of indirect effects on dental anxiety through rational thinking for the other FFMQ factors (which were not significant predictors of dental anxiety) was undertaken (see Supplementary Materials). This showed that Observing (effect: − 0.07, 95% CI = − 0.14 to − 0.02), Describing (effect: − 0.07, 95% CI = − 0.13 to − 0.02) and Non-judging (effect: − 0.02, 95% CI = − 0.07 to − 0.01) each had a significant indirect effect on dental anxiety through rational thinking. The indirect effect of Non-reacting through rational thinking was not significant (95% CI = − 0.07 to 0.02).

### Discussion

Study 2 showed that individuals with higher mindfulness had lower dental anxiety. We also found that all facets of mindfulness except Non-reacting and Nonjudging were positively correlated with rational thinking. Mediation analysis showed that rational thinking mediates the relationship between mindfulness and dental anxiety. People who are more mindful may think more rationally, and this is associated with having less dental anxiety.

Only Acting with Awareness was significantly negatively correlated with dental anxiety, although with a small correlation coefficient. Further forced entry multiple regression including all five factors showed that none of the mindfulness subscales were significant predictors of dental anxiety. Considering that mindfulness in general is a significant predictor, the result suggests that the effect of mindfulness on trait dental anxiety is mainly due to the overall mindfulness level rather than relying on specific facets of mindfulness. Bootstrapping analysis showed a significant indirect effect of mindfulness (total FFMQ score) on dental anxiety through rational thinking. Also, although the main effects of Observing, Describing, and Non-judging on dental anxiety were not significant, these three factors each had significant indirect effects on dental anxiety through rational thinking.

## General discussion

Previous research on the relationship between mindfulness and dental anxiety is limited and the results have shown some inconsistency, with Vaught^40^ finding that trait mindfulness measured by the MAAS was not related to dental anxiety, while Tellez et al.^41^ found that only the two facets of Acting with Awareness and Non-judging measured by the FFMQ were negatively correlated with dental anxiety. Our two studies, across two different samples, found that overall trait mindfulness measured by FFMQ is negatively correlated with both state dental anxiety and trait dental anxiety after controlling age and gender.

We also found that rational thinking mediates the relationship between mindfulness and dental anxiety. Consistent with previous research showing a significant positive correlation between mindfulness and rational thinking (r = 0.56)^50,51^ our research also demonstrated a medium/strong correlation between mindfulness and rational thinking (r = 0.43), suggesting mindful people are more likely to think rationally. In further analysis into each facet of mindfulness, consistent with Farrar et al.’s^50^ research, we also found that Observing, Describing and Acting with Awareness were positively correlated with rational thinking. However, the findings on Non-judging and Non-reacting were inconsistent. Non-reacting was significantly positively correlated with rationality according to Farrar et al.^51^, however, in our research it was not. Non-judging, was not a significant predictor in Farrar et al.’s^51^ research, but in the current research the regression analysis revealed a significant positive effect of Non-judging on rational thinking after controlling other facets of mindfulness. Research could further investigate the relationship between different facets of mindfulness and rational thinking.

We also explored the role each facet of mindfulness plays in the relationship between mindfulness and dental anxiety. Facet-level correlations with dental anxiety were inconsistent between study 1 and study 2. In study 1, each facet of mindfulness except Non-judging was negatively correlated with state dental anxiety with Acting with Awareness being the strongest, while in study 2, only Acting with Awareness significantly negatively correlated with trait dental anxiety, although with a small coefficient. The difference might be due to the sample variation or difference for state compared to trait measures of dental anxiety. Further research needs to be done to test the cause for the difference. Acting with Awareness showed the largest negative association with both state dental anxiety and trait dental anxiety, while Observing and Describing showed weak negative associations with state dental anxiety. This is consistent not only with Tellez et al.’s^41^ research on dental anxiety, but also with other types of anxiety^32,70,71^, demonstrating the significant role Acting with Awareness may play in mindfulness’ potential alleviation effect on anxiety. Non-reacting ranked the second largest in terms of the negative association with state dental anxiety and trait dental anxiety in correlation analysis and the regression analysis showed that it was still a significant predictor of state dental anxiety after controlling the other four facets. This is not consistent with previous research on dental anxiety^68^ and other types of anxiety^32,70,71^ showing that Non-reacting often has the weakest negative association with anxiety among the five factors of mindfulness. Non-judging was not significantly correlated with either state dental anxiety or trait dental anxiety in our studies. However, many previous studies have found that individuals who tend to adopt a non-judgmental stance towards their own thoughts and feelings have lower anxiety^32,41,70,71^ and Barcaccia et al.’s^70^ empirical research showed that nonjudgemental attitude is the strongest predictor of anxiety among all five factors. These differences for Non-reacting and Non-judging may be attributed to the specificity of dental anxiety. Dental anxiety is the reaction to physical pain and potential bodily harm caused by dental service and this reaction may remain whether you judge the experience as good or bad, as this reaction is very instinct-based and related with physical feelings, while general anxiety is related with neuroticism^72^ and the stress caused by individuals’ maladaptive patterns of thinking, judging, and responding^73^. This difference for Non-reacting and Non-judging between dental anxiety and general anxiety further shows that even though mindfulness has been shown in many studies to be strongly associated with general anxiety, it is worthwhile to extend the research to specific types of anxiety. Future research could further investigate the relationship between mindfulness and other specific types of anxiety, such as health anxiety and social anxiety.

The current research also has practical implications. The results highlighted that mindfulness is not only associated with general anxiety, but also dental anxiety specifically. So, clinical practitioners treating dental anxiety problems could identify the patients with low mindfulness (e.g., without meditation app use experience) and provide them more emotional comfort. Dental clinics are also encouraged to incorporate mindfulness interventions that are available to them and have been previously found effective in improving level of trait mindfulness, for example the short mindfulness-based technique designed by Whitaker^37^ for dental treatment and MBSR program that had better long-term effects on reducing dental anxiety according to research of Li et al.^38^. The relationship between rational thinking and dental anxiety also implies that to work with emotional distress, clinical practitioners should not only work with patients’ emotions, but also their thinking style. To promote rational thinking among patients may help to relieve them from emotional distress as well.

Our research has some limitations. First, as our sample was college students, the age range was narrow, limiting our results' applicability to different populations. Furthermore, the age-related correlations of the constructs assessed in this study should be carefully interpreted due to the small age range. Future research should aim to replicate this study in a more diverse sample to evaluate the generality of our findings. Also, although there were some respondents from other Asian countries, the number of the participants was limited. Future research could further investigate the relationship with samples in other countries. A second limitation with the current research is that although state and trait measures of dental anxiety were used, only trait measures of mindfulness and rational thinking were included. So further research may more precisely assess the inter-relationships between these variables at both the state and trait levels. A third limitation with the current research is that both of the studies had a cross-sectional design, thus cause-effect conclusions cannot be drawn. So, while the mediation analysis in the current research indicates plausible models, consistent with previous findings on mindfulness, rational thinking, and dental anxiety, further research is needed to establish if these models involve causal relationships. Also, further research is required to more fully investigate the relative influences of the different facets of mindfulness, and other possible mediating and moderating factors that may be involved in their relationships with rational thinking and with dental anxiety. In addition, the current research utilized self-report measures, which involve limitations such as the possibility of socially desirable responding. Intervention studies may test whether increasing aspects of mindfulness increases rational thinking, and whether increasing rational thinking decreases dental anxiety. Previous research on how to alleviate dental anxiety has examined medical interventions and psychological interventions such as cognitive behavior therapy^9,25^, hypnosis^18^, desensitization therapy^19^, etc. Our results suggest that mindfulness training may potentially help alleviate patients’ dental anxiety. Previous research on the effects of mindfulness interventions on dental anxiety has focused on designing techniques (e.g., Whitaker^37^), or a specific dental treatment (e.g. dental implant, in Li et al.^38^), or a particular group (e.g. children, in Taghvaei and Jahangiri^39^). Future research could empirically test the effects of mindfulness interventions on the wider population and for general dental anxiety.

## Conclusion

The main purpose of this study was to test the associations between mindfulness and dental anxiety. The initial hypotheses were verified by two studies. The first study used a scenario questionnaire, finding that mindfulness has a significant negative association with state dental anxiety. The second study found that mindfulness also has a negative association with trait dental anxiety, although, there was some inconsistency in bivariate correlations at the facet-level of mindfulness between the two studies. Furthermore, in Study 2 we also found that rational thinking positively correlated with mindfulness, and negatively correlated with dental anxiety, and that the negative effect of mindfulness on dental anxiety is mediated by rational thinking. These results may inform interventions to deal with dental anxiety.

## Supplementary Information

Supplementary Information.

## Data Availability

The data are available from the corresponding author on request.

## References

[1] Roy J, Dempster LJ (2018). Dental anxiety associated with orthodontic care: Prevalence and contributing factors. Semin. Orthod..

[2] Carlsson V, Hakeberg M, Wide Boman U (2015). Associations between dental anxiety, sense of coherence, oral health-related quality of life and health behaviour—A national Swedish cross-sectional survey. BMC Oral Health.

[3] Weinstein P, Shimono T, Domoto P, Wohlers K, Matsumura S, Ohmura M, Omachi K (1992). Dental fear in Japan: Okayama Prefecture school study of adolescents and adults. Anesth. Prog..

[4] Stouthard ME, Hoogstraten J (1990). Prevalence of dental anxiety in the Netherlands. Community Dent. Oral Epidemiol..

[5] Ozlek E, Yıldırım A, Koc A, Boysan M (2019). Socio-demographic determinants of dental anxiety and fear among college students. East. J. Med..

[6] Bäck K, Hakeberg M, Wide U, Hange D, Dahlström L (2020). Orofacial pain and its relationship with oral health-related quality of life and psychological distress in middle-aged women. Acta Odontol. Scand..

[7] Svensson L, Hakeberg M, Wide U (2020). Evaluating the validity of the Index of Dental Anxiety and Fear (IDAF-4C+) in adults with severe dental anxiety. Eur. J. Oral Sci..

[8] Humphris GM, Morrison T, Lindsay SJE (1995). The Modified Dental Anxiety Scale: Validation and United Kingdom norms. Community Dent. Health.

[9] Appukuttan DP (2016). Strategies to manage patients with dental anxiety and dental phobia: Literature review. Clin. Cosmet. Investig. Dent..

[10] Gayathri PS, Krithika C, Kumar A, Selvarathi K, Christy Jospeh Samuel K, Manju J (2021). Awareness about management of pain and anxiety during dental treatments among dental students. Indian J. Forensic Med. Toxicol..

[11] Walsh LJ (2007). Dental anxiety: Causes, complications and management approaches. Int. Dent. SA Australas. Ed..

[12] Selvi VT, Devi RG, Jothipriya A (2020). Prevalence of dental anxiety among the OP patients in Saveetha Dental College. Drug Invent. Today.

[13] Armfield JM (2010). Development and psychometric evaluation of the Index of Dental Anxiety and Fear (IDAF-4C+). Psychol. Assess..

[14] Haddad DF (2018). Pain-reducing techniques for delivery of dental anesthesia. Dent. Acad. Contin. Educ..

[15] Allan FK, Peckham E, Liu J, Dietz KC, Zhang T, Arakaki A, MacPherson H (2018). Acupuncture for anxiety in dental patients: Systematic review and meta-analysis. Eur. J. Integr. Med..

[16] Morarend QA, Spector ML, Dawson DV, Clark SH, Holmes DC (2011). The use of a respiratory rate biofeedback device to reduce dental anxiety: An exploratory investigation. Appl. Psychophysiol. Biofeedback.

[17] Shahnavaz S, Hedman E, Grindefjord M, Reuterskiöld L, Dahllöf G (2016). Cognitive behavioral therapy for children with dental anxiety: A randomized controlled trial. JDR Clin. Transl. Res..

[18] Griffiths MJ (2017). The role of hypnotherapy in evidence-based clinical practice. Oral Dis..

[19] Doering S, Ohlmeier MC, Jongh A, Hofmann A, Bisping V (2013). Efficacy of a trauma-focused treatment approach for dental phobia: A randomized clinical trial. Eur. J. Oral Sci..

[20] Ainscough SL, Windsor L, Tahmassebi JF (2019). A review of the effect of music on dental anxiety in children. Eur. Arch. Paediatr. Dent..

[21] Premkumar KS, Aafaque S, Sumalatha S, Narendran N (2019). Effect of aromatherapy on dental anxiety among orthodontic patients: A randomized controlled trial. Cureus.

[22] Kabat-Zinn J (1982). An outpatient program in behavioral medicine for chronic pain patients based on the practice of mindfulness meditation: Theoretical considerations and preliminary results. Gen. Hosp. Psychiatry.

[23] Kabat-Zinn J (1994). Wherever You Go, There You Are: Mindfulness Meditation for Everyday life.

[24] Baer RA, Smith GT, Hopkins J, Krietemeyer J, Toney L (2006). Using self-report assessment methods to explore facets of mindfulness. Assessment.

[25] Sharma PK, Kumra R (2022). Relationship between mindfulness, depression, anxiety and stress: Mediating role of self-efficacy. Personal. Individ. Differ..

[26] Dunn C, Hanieh E, Roberts R, Powrie R (2012). Mindful pregnancy and childbirth: Effects of a mindfulness-based intervention on women's psychological distress and well-being in the perinatal period. Arch. Womens Ment. Health.

[27] Bossi F, Zaninotto F, D'Arcangelo S, Lattanzi N, Malizia AP, Ricciardi E (2022). Mindfulness-based online intervention increases well-being and decreases stress after Covid-19 lockdown. Sci. Rep..

[28] Fazia T, Bubbico F, Berzuini G, Tezza LD, Cortellini C, Bruno S, Bernardinelli L (2021). Mindfulness meditation training in an occupational setting: Effects of a 12-weeks mindfulness-based intervention on wellbeing. Work (Reading, Mass).

[29] Hofmann SG, Gómez AF (2017). Mindfulness-based interventions for anxiety and depression. Psychiatr. Clin..

[30] Grossman P, Niemann L, Schmidt S, Walach H (2004). Mindfulness-based stress reduction and health benefits: A meta-analysis. J. Psychosom. Res..

[31] Khoury B, Lecomte T, Fortin G, Masse M, Therien P, Bouchard V, Chapleau M, Paquin K, Hofmann SG (2013). Mindfulness-based therapy: A comprehensive meta-analysis. Clin. Psychol. Rev..

[32] Hambour VK, Zimmer-Gembeck MJ, Clear S, Rowe S, Avdagic E (2018). Emotion regulation and mindfulness in adolescents: Conceptual and empirical connection and associations with social anxiety symptoms. Personal. Individ. Differ..

[33] David A, Rubinsten O, Berkovich-Ohana A (2022). Math anxiety, self-centeredness, and dispositional mindfulness. J. Educ. Psychol..

[34] Kabat-Zinn J (2005). Bringing mindfulness to medicine. Altern. Ther. Health Med..

[35] Brown KW, Ryan RM (2003). The benefits of being present: Mindfulness and its role in psychological well-being. J. Pers. Soc. Psychol..

[36] Lengacher CA, Johnson-Mallard V, Post-White J, Moscoso MS, Jacobsen PB, Klein TW, Widen RH, Fitzgerald SG, Shelton MM, Barta M, Goodman M, Cox CE, Kip KE (2009). Randomized controlled trial of mindfulness-based stress reduction (MBSR) for survivors of breast cancer. Psychooncology.

[37] Whitaker, D. Distraction, mindfulness and pain: Acceptance-based approach to dental fear and anxiety*.* https://static1.squarespace.com/static/54fdb217e4b0edea64907af7/t/5527afd8e4b0c85c2c9cbb94/1428664280466/%C3%85rskursus2015_Distraction_Notes%26Handouts__2015.pdf (2015).

[38] Li N, Li M, Wen B, Jiang L, Huang Y, Chen X, Shuai B, Zhang Q (2018). Effects of mindfulness-based stress reduction and music therapy on anxiety and pain in dental anxiety of implant teeth patients. Nurs. J. Chin. People's Lib. Army..

[39] Taghvaei D, Jahangiri MM (2016). The effectiveness mindfulness training on reducing of anxiety and pain intensity resulting from dentistry procedures in children. Iran. J. Pediatr. Dent..

[40] Vaught, D. T. Relation of dental anxiety and mindfulness: A comparison of pre-health and non pre-health students. Undergraduate Research Scholars Program. https://hdl.handle.net/1969.1/157649 (2017).

[41] Tellez M, Kinner DG, Heimberg RG, Lim S, Ismail AI (2015). Prevalence and correlates of dental anxiety in patients seeking dental care. Community Dent. Oral Epidemiol..

[42] McCluskey DL, Haliwa I, Wilson JM, Keeley JW, Shook NJ (2020). Experiential avoidance mediates the relation between mindfulness and anxiety. Curr. Psychol..

[43] Ostafin BD, Brooks JJ, Laitem M (2014). Affective reactivity mediates an inverse relation between mindfulness and anxiety. Mindfulness.

[44] McDonald HM, Sherman KA, Petocz P, Kangas M, Grant K, Kasparian NA (2016). Mindfulness and the experience of psychological distress: The mediating effects of emotion regulation and attachment anxiety. Mindfulness.

[45] Freudenthaler L, Turba JD, Tran US (2017). Emotion regulation mediates the associations of mindfulness on symptoms of depression and anxiety in the general population. Mindfulness.

[46] Parmentier FB, García-Toro M, García-Campayo J, Yañez AM, Andrés P, Gili M (2019). Mindfulness and symptoms of depression and anxiety in the general population: The mediating roles of worry, rumination, reappraisal and suppression. Front. Psychol..

[47] Epstein S (1990). Cognitive-Experiential Self-Theory. Handbook of Personality: Theory and Research.

[48] Epstein S, Pacini R, Denes-Raj V, Heier H (1996). Individual differences in intuitive–experiential and analytical–rational thinking styles. J. Pers. Soc. Psychol..

[49] Kang Y, Gruber J, Gray JR (2013). Mindfulness and de-automatization. Emot. Rev..

[50] Farrar S, Yarrow K, Tapper K (2020). The effect of mindfulness on cognitive reflection and reasoning. Mindfulness.

[51] Farrar S, Tapper DK (2018). The effect of mindfulness on rational thinking. Appetite.

[52] Kirk U, Downar J, Montague PR (2011). Interoception drives increased rational decision-making in meditators playing the ultimatum game. Front. Neurosci..

[53] Ellis A (1957). Rational psychotherapy and individual psychology. J. Individ. Psychol..

[54] Bowman AW, Turner MJ (2022). When time is of the essence: The use of rational emotive behavior therapy (REBT) informed single-session therapy (SST) to alleviate social and golf-specific anxiety, and improve wellbeing and performance, in amateur golfers. Psychol. Sport Exerc..

[55] Chamberlain JM, Haaga DA (2001). Unconditional self-acceptance and responses to negative feedback. J. Ration. Emot. Cogn. Behav. Ther..

[56] David D, Szentagotai A, Eva K, Macavei B (2005). A synopsis of rational-emotive behavior therapy (REBT); Fundamental and applied research. J. Ration. Emot. Cogn. Behav. Ther..

[57] Caserta DA, Dowd ET, David D, Ellis A, David D, Lynn SJ, Ellis A (2010). Rational and irrational beliefs in primary prevention and mental health. Rational and Irrational Beliefs: Research, Theory and Clinical Practice.

[58] Cohen J (1992). A power primer. Psychol. Bull..

[59] Hulley SB, Cummings SR, Browner WS, Grady D, Newman TB (2013). Designing Clinical Research: An Epidemiologic Approach.

[60] Deng Y, Liu X, Rodriguez MA, Xia C (2011). The five-facet mindfulness questionnaire: Psychometric properties of the Chinese version. Mindfulness.

[61] Banks K, Newman E, Saleem J (2015). An overview of the research on mindfulness-based interventions for treating symptoms of posttraumatic stress disorder: A systematic review. J. Clin. Psychol..

[62] Veehof MM, Ten Klooster PM, Taal E, Westerhof GJ, Bohlmeijer ET (2011). Psychometric properties of the Dutch Five Facet Mindfulness Questionnaire (FFMQ) in patients with fibromyalgia. Clin. Rheumatol..

[63] Le SH, Tonami K, Umemori S, Nguyen LB, Ngo LQ, Mataki S (2018). The potential of heart rate variability for exploring dental anxiety in mandibular third molar surgery. Int. J. Oral Maxillofac. Surg..

[64] Perpiñá-Galvañ J, Richart-Martínez M, Cabañero-Martínez MJ (2011). Reliability and validity of a short version of the STAI anxiety measurement scale in respiratory patients. Archivos de Bronconeumología (English Edition).

[65] He J (2021). Measurement invariance of Chinese version of State-Trait Anxiety Inventory Form. Chin. J. Clin. Psychol..

[66] Deogade SC, Suresan V (2016). Psychometric assessment of anxiety with the Modified Dental Anxiety Scale among central Indian adults seeking oral health care to a dental school. Ind. Psychiatry J..

[67] Ng SK, Stouthard ME, Keung Leung W (2015). Validation of a Chinese version of the dental anxiety inventory. Community Dent. Oral Epidemiol..

[68] Wen, Y. Individual differences of college students' Rational-Experiential thinking method and its relationship with social problem solving (Postgraduate Dissertation) (2012).

[69] Zhao X, Lynch JG, Chen Q (2015). Reconsidering Baron and Kenny: Myths and truths about mediation analysis. J. Consum. Res..

[70] Barcaccia B, Baiocco R, Pozza A, Pallini S, Mancini F, Salvati M (2019). The more you judge the worse you feel. A judgemental attitude towards one's inner experience predicts depression and anxiety. Person. Individ. Differ..

[71] Lee FK, Zelman DC (2019). Boredom proneness as a predictor of depression, anxiety and stress: The moderating effects of dispositional mindfulness. Person. Individ. Differ..

[72] Giluk TL (2009). Mindfulness, big give personality, and affect: A meta-analysis. Person. Individ. Differ..

[73] Hofmann SG, Asnaani A, Vonk IJ, Sawyer AT, Fang A (2012). The efficacy of cognitive behavioral therapy: A review of meta-analyses. Cogn. Ther. Res..

