# Supplementary material for: Rational thinking as a mediator of the relationship between mindfulness and dental anxiety
Source: Sci Rep. 2023 Feb 22;13:3104. doi: 10.1038/s41598-023-28879-4 (PMC9946927; doi:10.1038/s41598-023-28879-4)
Supplement: Supplementary file 1 — Supplementary Information. [file 41598_2023_28879_MOESM1_ESM.docx]

Mindfulness and Dental Anxiety: The Mediating Role of Rational Thinking

**Author:**

Jiaxuan Yao, Department of Social Statistics, The University of Manchester, HBS Building, Oxford Road, Manchester M13 9PL, United Kingdom. International Business School Suzhou, Xi’an Jiaotong-Liverpool University, No.8 Chongwen Road, Suzhou, China.

Richard Carciofo, Lecturer, School of Psychology and Clinical Language Sciences, University of Reading, Reading, United Kingdom.

ORCID: 0000-0003-2069-7047

Li (Sunny) Pan, Assistant Professor, International Business School Suzhou, Xi’an Jiaotong-Liverpool University, No.8 Chongwen Road, Suzhou, China. Sunny.pan@xjtlu.edu.cn; +86 (0) 512-8816 7128.

ORCID: 0000-0001-9291-3541

**Supplementary materials**

Table S1. Summary of sample demographic characteristics of study 1 (N=206).

| Demographic Characteristics | Frequency | Percentage |
| --- | --- | --- |
| Gender(male) | 108 | 52.40% |
| Age |  |  |
| 18 | 18 | 8.70% |
| 19 | 40 | 19.40% |
| 20 | 44 | 21.40% |
| 21 | 39 | 18.90% |
| 22 | 33 | 16% |
| 23 | 25 | 12.10% |
| 24 | 4 | 1.90% |
| 25 | 3 | 1.50% |
| Education Level |  |  |
| Junior college | 25 | 12.10% |
| Bachelor | 176 | 85.40% |
| Master | 5 | 2.40% |
| Monthly Living Expenses |  |  |
| <1,000RMB | 20 | 9.70% |
| 1,000-1,999RMB | 148 | 71.80% |
| 2,000-2,999RMB | 28 | 13.60% |
| 3,000-3,999RMB | 7 | 3.40% |
| 4,000-4,999RMB | 2 | 1% |
| >5,000RMB | 1 | 0.50% |

Table S2. Summary of sample demographic characteristics of study 2 (N=396).

| Demographic Characteristics | Frequency | Percentage |
| --- | --- | --- |
| Gender(male) | 170 | 43.1% |
| Age |  |  |
| 16 | 3 | .8% |
| 17 | 4 | 1.0% |
| 18 | 192 | 48.7% |
| 19 | 163 | 41.4% |
| 20 | 21 | 5.3% |
| 21 | 4 | 1.0% |
| 22 | 3 | .8% |
| 23 | 2 | .5% |
| 25 | 1 | .3% |
| 26 | 1 | .3% |
| Nationality |  |  |
| China | 362 | 91.9% |
| Korea | 14 | 3.6% |
| Indonesia | 10 | 2.5% |
| Other | 8 | 2.0% |

**Table S3. Study 2 results for mindfulness- Observing**

|  | DV=Dental Anxiety | DV= Rational thinking (Mediator) | DV= Dental Anxiety  (Full model) |
| --- | --- | --- | --- |
| Gender | .128* | -.038 | .121* |
| Age | -.031 | -.036 | -.038 |
| Observing | −.023 | .268** | .025 |
| Rational thinking |  |  | −.177** |
| R^2^ | .021 | .065 | .039 |
| F | 2.535 | 10.099 | 4.935 |

Note: *p<.05, **p<.01.

**Table S4.** Mediation results for mindfulness **- Observing**

|  | Effect | SE | LLCI | ULCI | Effect Ratio |
| --- | --- | --- | --- | --- | --- |
| Total effect | -.0350 | .0774 | -.1872 | -.1172 |  |
| Direct effect | .0379 | .0792 | -.1179 | .1936 | -108.29% |
| Indirect effect | -.0718 | .0294 | -.1390 | -.0241 | 205.14% |

**Table S5. Study 2 results for mindfulness- Describing**

|  | DV=Dental Anxiety | DV= Rational thinking (Mediator) | DV= Dental Anxiety  (Full model) |
| --- | --- | --- | --- |
| Gender | .129* | -.040 | .122* |
| Age | -.025 | -.039 | -.031 |
| Describing | −.083 | .308** | -.033 |
| Rational thinking |  |  | −.161** |
| R^2^ | .025 | .095 | .049 |
| F | 3.380 | 13.607 | 4.980 |

Note: *p<.05, **p<.01.

**Table S6.** Mediation results for mindfulness **- Describing**

|  | Effect | SE | LLCI | ULCI | Effect Ratio |
| --- | --- | --- | --- | --- | --- |
| Total effect | -.1184 | .0722 | -.2603 | -.0235 |  |
| Direct effect | -.0475 | .0750 | -.1949 | .0999 | 40.12% |
| Indirect effect | -.0699 | .0267 | -.1271 | -.0228 | 59.04% |

**Table S7. Study 2 results for mindfulness- Acting with awareness**

|  | DV=Dental Anxiety | DV= Rational thinking (Mediator) | DV= Dental Anxiety  (Full model) |
| --- | --- | --- | --- |
| Gender | .135** | -.058 | .127* |
| Age | -.022 | -.038 | -.028 |
| Acting with awareness | −.119* | .339** | -.069 |
| Rational thinking |  |  | −.148** |
| R^2^ | .033 | .115 | .052 |
| F | 4.383 | 16.909 | 5.323 |

Note: *p<.05, **p<.01.

**Table S8.** Mediation results for mindfulness **- Acting with awareness**

|  | Effect | SE | LLCI | ULCI | Effect Ratio |
| --- | --- | --- | --- | --- | --- |
| Total effect | -.0745 | .1003 | -.2718 | -.1228 |  |
| Direct effect | -.0585 | .0991 | -.2533 | .1364 | 78.52% |
| Indirect effect | -.0160 | .0241 | -.0758 | .0227 | 21.48% |

**Table S9. Study 2 results for mindfulness- Nonjudging**

|  | DV=Dental Anxiety | DV= Rational thinking (Mediator) | DV= Dental Anxiety  (Full model) |
| --- | --- | --- | --- |
| Gender | .135** | -.043 | .127* |
| Age | -.031 | -.007 | -.032 |
| Nonjudging | −.076 | .068 | -.065 |
| Rational thinking |  |  | −.166** |
| R^2^ | .024 | .006 | .052 |
| F | 3.245 | .777 | 5.320 |

Note: *p<.05, **p<.01.

**Table S10.** Mediation results for mindfulness **- Nonjudging**

|  | Effect | SE | LLCI | ULCI | Effect Ratio |
| --- | --- | --- | --- | --- | --- |
| Total effect | -.1335 | .0882 | -.3070 | -.0399 |  |
| Direct effect | -.1135 | .0873 | -.2851 | .0580 | 85.02% |
| Indirect effect | -.0200 | .0216 | -.0741 | -.0143 | 14.98% |

**Table S11. Study 2 results for mindfulness- Nonreactivity**

|  | DV=Dental Anxiety | DV= Rational thinking (Mediator) | DV= Dental Anxiety  (Full model) |
| --- | --- | --- | --- |
| Gender | .125* | -.033 | .120* |
| Age | -.031 | -.007 | -.033 |
| Nonreactivity | −.037 | .048 | -.029 |
| Rational thinking |  |  | −.169** |
| R^2^ | .020 | .004 | .049 |
| F | 2.653 | .461 | 4.967 |

Note: *p<.05, **p<.01.

**Table S12.** Mediation results for mindfulness **- Nonreactivity**

|  | Effect | SE | LLCI | ULCI | Effect Ratio |
| --- | --- | --- | --- | --- | --- |
| Total effect | -.0745 | .1003 | -.2718 | .1228 |  |
| Direct effect | -.0585 | .0991 | -.2533 | .1364 | 78.52% |
| Indirect effect | -.0160 | .0241 | -.0758 | .0227 | 21.48% |

**Questionnaire**

**1. Study 1**

Dear respondents, you are welcome to participate in this study. This study is aimed at college students at least 18 years old, and you will be required to complete an online survey that will take about 10 minutes. Your response will be treated as confidential, and we will use our best reasonable efforts to prevent disclosure of your information. Your participation in this study is voluntary. You may not participate in the study, or if you decide to participate, you may later change your mind and withdraw from the study. You may exit at any time without answering any questions. 亲爱的伙伴，欢迎您参与到本次研究中。本研究主要针对18岁以上的大学生群体，您将需要完成一个在线调查，大约需要10分钟。您的答复将被视为机密，我们将作出最大合理的努力，以防止您信息的泄漏。您参与本项研究是自愿的。您可以不参加这项研究，如果您决定参加，您之后可以改变主意并退出研究。您可以在任何时间不回答任何问题或退出。

(1). Please rate each of the following statements with the number that best describes your own opinion of what is generally true for you. 请根据最近几周的情况来考虑每个条目，请如实地凭着您的直觉去选择答案。 这些答案没有所谓的“对”与“错”、“好”与“坏”之分。我们最希望得到的是您自己真正的体验。

| Please rate each of the following statements with the number that best describes your own opinion of what is generally true for you. | | Never or very rarely true =1  不符合 | Rarely true  =2  较不符合 | Sometimes true =3  不确定 | Often true =4  较符合 | Very often or always  True  =5  完全符合 |
| --- | --- | --- | --- | --- | --- | --- |
| 1 | When I'm walking, I deliberately notice the sensations of my body moving. 在行走时，我会有意关注身体部位在行进中的感觉。 |  |  |  |  |  |
| 2 | I'm good at finding words to describe my feelings. 我擅长于用言语描述我的情感。 |  |  |  |  |  |
| 3 | I criticize myself for having irrational or inappropriate emotions. 我为自己有不理智的情绪或不合适的情绪而责备自己。 |  |  |  |  |  |
| 4 | 我感受到了我的情绪和情感，但我不必对它们做出反应。I perceive my feelings and emotions without having to react to them. |  |  |  |  |  |
| 5 | When I do things, my mind wanders off and I’m easily distracted. 在做事的时候，我经常走神，而且很容易被干扰。 |  |  |  |  |  |
| 6 | When I take a shower or bath, I stay alert to the sensations of water on my body. 在洗澡时，我会留心于水淌过身体的感觉。 |  |  |  |  |  |
| 7 | I can easily put my beliefs, opinions, and expectations into words. 我能清晰表达自己的信念、观点以及期望。 |  |  |  |  |  |
| 8 | I don’t pay attention to what I’m doing because I’m daydreaming, worrying, or otherwise distracted. 我没有注意到我在做什么事情，这是因为我在做白日梦， 在担忧或分心于外界。 |  |  |  |  |  |
| 9 | I watch my feelings without getting lost in them. 我观察自己的情绪，而不迷失其中。 |  |  |  |  |  |
| 10 | I tell myself I shouldn’t be feeling the way I’m feeling. 我告诉自己，我不应该以我现在的这种方式来感受此时的情感。 |  |  |  |  |  |
| 11 | I notice how foods and drinks affect my thoughts, bodily sensations, and emotions. 我感受到食物和饮料是如何影响着我的想法，身体的感受和情绪。 |  |  |  |  |  |
| 12 | It’s hard for me to find the words to describe what I’m thinking. 我难以找到词语来表达我的所思所想。 |  |  |  |  |  |
| 13 | I am easily distracted. 我很容易分心。 |  |  |  |  |  |
| 14 | I believe some of my thoughts are abnormal or bad and I shouldn’t think that way. 我认为我的一些想法是不好的，是不该存在的。 |  |  |  |  |  |
| 15 | I pay attention to sensations, such as the wind in my hair or sun on my face. 我会注意我的一些感觉，比如微风拂过脸庞。 |  |  |  |  |  |
| 16 | I have trouble thinking of the right words to express how I feel about things. 我很难用合适的语言来表达我的感受。 |  |  |  |  |  |
| 17 | I make judgments about whether my thoughts are good or bad. 我会评判自己的想法是好是坏。 |  |  |  |  |  |
| 18 | I find it difficult to stay focused on what’s happening in the present. 我难以把注意力集中在当前发生的事情上。 |  |  |  |  |  |
| 19 | When I have distressing thoughts or images, I “step back” and am aware of the thought or image without getting taken over by it. 当我有悲伤的想法或景象时，我会“退一步”， 并去觉知那些想法或景象的存在而不被其控制。 |  |  |  |  |  |
| 20 | I pay attention to sounds, such as clocks ticking, birds chirping, or cars passing. 我会注意一些声音，比如：时钟的滴答。 |  |  |  |  |  |
| 21 | In difficult situations, I can pause without immediately reacting. 在困难的情境下，我会暂停一下，不马上做出反应。 |  |  |  |  |  |
| 22 | When I have a sensation in my body, it’s difficult for me to describe it because I can’t find the right words. 当我的身体有种感觉时，我很难用合适的词来描述它。 |  |  |  |  |  |
| 23 | It seems I am “running on automatic” without much awareness of what I’m doing. 我好像是自动在做一下事情，并没有完全意识到它。 |  |  |  |  |  |
| 24 | When I have distressing thoughts or images, I feel calm soon after. 通常，当我有令人伤感的想法或者景象，能很快恢复平静。 |  |  |  |  |  |
| 25 | I tell myself that I shouldn’t be thinking the way I’m thinking. 我告诉自己，不该思考我此刻正在思考的事情。 |  |  |  |  |  |
| 26 | I notice the smells and aromas of things. 我闻到了周围一些东西的气味或者芳香。 |  |  |  |  |  |
| 27 | Even when I’m feeling terribly upset, I can find a way to put it into words. 即使是非常不安的时候，我也能找到词语来表达它。 |  |  |  |  |  |
| 28 | I rush through activities without being really attentive to them. 我草草地做完一些事情，没有真正集中注意力。 |  |  |  |  |  |
| 29 | When I have distressing thoughts or images, I am able just to notice them without reacting. 当有烦恼情绪时，我能做到只是去注意，而不做出相应反应。 |  |  |  |  |  |
| 30 | I think some of my emotions are bad or inappropriate and I shouldn’t feel them. 我想有些情绪是不对或者不合适的，我不应该体验到他们。 |  |  |  |  |  |
| 31 | I notice visual elements in art or nature, such as colors, shapes, textures, or patterns of light and shadow. 我注意到艺术品和自然事物中一些视觉元素，如颜色形状光影。 |  |  |  |  |  |
| 32 | My natural tendency is to put my experiences into words. 我总是倾向于用词语来描述我的体验。 |  |  |  |  |  |
| 33 | When I have distressing thoughts or images, I just notice them and let them go. 当我有令人痛苦的想法或景象时，我通常只是去注意它，顺其自然。 |  |  |  |  |  |
| 34 | I do jobs or tasks automatically without being aware of what I’m doing. 我总是自动地工作或完成某项任务，而没有意识到我在做什么。 |  |  |  |  |  |
| 35 | When I have distressing thoughts or images, I judge myself as good or bad depending what the thought or image is about. 通常我有令人困扰的想法或景象时，我会根据当时脑中所想来判断自己是对是错。 |  |  |  |  |  |
| 36 | I pay attention to how my emotions affect my thoughts and behavior. 我会去注意，我的情绪是如何影响我的想法和行为的。 |  |  |  |  |  |
| 37 | I can usually describe how I feel at the moment in considerable detail. 我通常能特别详细地描述出我此刻的感觉。 |  |  |  |  |  |
| 38 | I find myself doing things without paying attention. 我发现自己做事情的时候，不专心在所做的事上。 |  |  |  |  |  |
| 39 | I disapprove of myself when I have irrational ideas. 当不理智的想法出现，我会自我否决。 |  |  |  |  |  |

(2). 接下来，请您仔细阅读并想象下面的场景 please read carefully and imagine the following scenario:


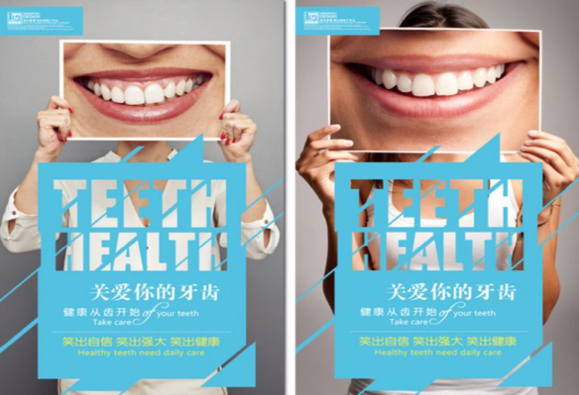


Today is International Dental Day, and you see the desk of a private dental hospital promoting a 'free oral examination' campaign at the subway station. You recently just consider orthodontics, so you went to the consultation. After consulting with your doctor for a preliminary oral examination, you can use the Hidden Beauty Invisible Dental Appliance depending on your dental condition, a new type of dental appliance that is nearly invisible to wear and more comfortable than other appliances, also, it can effectively maintain the patient's oral hygiene. The entire course of orthodontic treatment takes 12 to 18 months, during which time you will need to have regular follow-up appointments. But due to the alignment of your teeth, it may be necessary to remove one tooth first, and the final corrective regimen will need to be further examined before it can be determined. After the correction is over, your teeth will become neater and more beautiful.

今天是国际爱牙日，你在地铁站看到私立牙科医院服务台正在宣传‘免费口腔检查’的活动，正好你最近有考虑进行牙齿矫正，于是你上前去咨询。咨询医生对你进行了初步的口腔检查之后告诉你，根据你的牙齿情况，你可以使用隐适美隐形牙齿矫正器，这种新型的牙齿矫正器的佩戴效果近乎隐形，并且相比其他矫正器更加舒适，可有效保持患者的口腔卫生。整个矫正疗程需要12个月-18个月，在此期间你需要定期复诊，但是由于你牙齿排列情况，可能需要先拔掉一颗牙，最终矫正方案需要进一步检查后才能确定。当矫正结束时，你的牙齿会变得更加整齐和美观。

Read each statement and then choose the appropriate number to the right of the statement to indicate how you feel now. Do not spend too much time on any one statement. 请就你此时此刻的感觉选择最能表现你状态的陈述，不用过多思考。

| Please rate each of the following statements with the number that best describes your own opinion of what is generally true for you. | | Not at all =1  完全没有 | Some what  =2  有一些 | Moderately so=3  比较符合 | Very much so =4  非常符合 |
| --- | --- | --- | --- | --- | --- |
| 1 | I feel calm 我感到心情平静 |  |  |  |  |
| 2 | I feel relax 我感到放松 |  |  |  |  |
| 3 | I feel sacred 我感到害怕 |  |  |  |  |
| 4 | I feel comfortable 我感到舒适 |  |  |  |  |
| 5 | I feel tense 我感到紧张 |  |  |  |  |
| 6 | I feel worry 我感到担心 |  |  |  |  |
| 7 | I feel happy 我感到愉快 |  |  |  |  |

(3). Your age您的年龄:____________________

(4). Your gender您的性别:

○Male男 ○Female女

(5). Your educational level您的受教育程度:

○Junior college 大专 ○Bachelor 本科 ○Master 硕士

(6). Your monthly cost of living 您的月消费：

○<1,000RMB ○1,000-1,999RMB ○2,000-2,999RMB ○3,000-3,999RMB ○4,000-4,999RMB ○>5,000RMB

**2. Study 2**

This survey is part of MAN008 Individual Report. Please read the questions and choose the answer that best describe how you feel. There is no right answer. As long as you finish the survey, you will receive 5 marks. But there is an attention test question in the survey. If you don't read the questions and just choose randomly, you will fail this test and would not be able to receive the 5 marks.  Your answers in the survey will be kept confidential. 本问卷是MAN008课程个人作业的一部分。请在认真阅读题目后凭直觉回答，不需要进行过多思考，答案答案没有对错，填写完成即可以得到5分。但问卷中会有注意力测试题，如果不阅读题目随便作答，则不能通过测试，将不能获得5分。你的答案会得到严格保密。

(1). Please rate each of the following statements with the number that best describes your own opinion of what is generally true for you. 请根据最近几周的情况来考虑每个条目，请如实地凭着您的直觉去选择答案。 这些答案没有所谓的“对”与“错”、“好”与“坏”之分。我们最希望得到的是您自己真正的体验。

| Please rate each of the following statements with the number that best describes your own opinion of what is generally true for you. | | Never or very rarely true =1  不符合 | Rarely true  =2  较不符合 | Sometimes true =3  不确定 | Often true =4  较符合 | Very often or always  True  =5  完全符合 |
| --- | --- | --- | --- | --- | --- | --- |
| 1 | When I'm walking, I deliberately notice the sensations of my body moving. 在行走时，我会有意关注身体部位在行进中的感觉。 |  |  |  |  |  |
| 2 | I'm good at finding words to describe my feelings. 我擅长于用言语描述我的情感。 |  |  |  |  |  |
| 3 | I criticize myself for having irrational or inappropriate emotions. 我为自己有不理智的情绪或不合适的情绪而责备自己。 |  |  |  |  |  |
| 4 | 我感受到了我的情绪和情感，但我不必对它们做出反应。I perceive my feelings and emotions without having to react to them. |  |  |  |  |  |
| 5 | When I do things, my mind wanders off and I’m easily distracted. 在做事的时候，我经常走神，而且很容易被干扰。 |  |  |  |  |  |
| 6 | When I take a shower or bath, I stay alert to the sensations of water on my body. 在洗澡时，我会留心于水淌过身体的感觉。 |  |  |  |  |  |
| 7 | I can easily put my beliefs, opinions, and expectations into words. 我能清晰表达自己的信念、观点以及期望。 |  |  |  |  |  |
| 8 | I don’t pay attention to what I’m doing because I’m daydreaming, worrying, or otherwise distracted. 我没有注意到我在做什么事情，这是因为我在做白日梦， 在担忧或分心于外界。 |  |  |  |  |  |
| 9 | I watch my feelings without getting lost in them. 我观察自己的情绪，而不迷失其中。 |  |  |  |  |  |
| 10 | I tell myself I shouldn’t be feeling the way I’m feeling. 我告诉自己，我不应该以我现在的这种方式来感受此时的情感。 |  |  |  |  |  |
| 11 | I notice how foods and drinks affect my thoughts, bodily sensations, and emotions. 我感受到食物和饮料是如何影响着我的想法，身体的感受和情绪。 |  |  |  |  |  |
| 12 | It’s hard for me to find the words to describe what I’m thinking. 我难以找到词语来表达我的所思所想。 |  |  |  |  |  |
| 13 | I am easily distracted. 我很容易分心。 |  |  |  |  |  |
| 14 | I believe some of my thoughts are abnormal or bad and I shouldn’t think that way. 我认为我的一些想法是不好的，是不该存在的。 |  |  |  |  |  |
| 15 | I pay attention to sensations, such as the wind in my hair or sun on my face. 我会注意我的一些感觉，比如微风拂过脸庞。 |  |  |  |  |  |
| 16 | I have trouble thinking of the right words to express how I feel about things. 我很难用合适的语言来表达我的感受。 |  |  |  |  |  |
| 17 | I make judgments about whether my thoughts are good or bad. 我会评判自己的想法是好是坏。 |  |  |  |  |  |
| 18 | I find it difficult to stay focused on what’s happening in the present. 我难以把注意力集中在当前发生的事情上。 |  |  |  |  |  |
| 19 | When I have distressing thoughts or images, I “step back” and am aware of the thought or image without getting taken over by it. 当我有悲伤的想法或景象时，我会“退一步”， 并去觉知那些想法或景象的存在而不被其控制。 |  |  |  |  |  |
| 20 | I pay attention to sounds, such as clocks ticking, birds chirping, or cars passing. 我会注意一些声音，比如：时钟的滴答。 |  |  |  |  |  |
| 21 | In difficult situations, I can pause without immediately reacting. 在困难的情境下，我会暂停一下，不马上做出反应。 |  |  |  |  |  |
| 22 | When I have a sensation in my body, it’s difficult for me to describe it because I can’t find the right words. 当我的身体有种感觉时，我很难用合适的词来描述它。 |  |  |  |  |  |
| 23 | It seems I am “running on automatic” without much awareness of what I’m doing. 我好像是自动在做一下事情，并没有完全意识到它。 |  |  |  |  |  |
| 24 | When I have distressing thoughts or images, I feel calm soon after. 通常，当我有令人伤感的想法或者景象，能很快恢复平静。 |  |  |  |  |  |
| 25 | I tell myself that I shouldn’t be thinking the way I’m thinking. 我告诉自己，不该思考我此刻正在思考的事情。 |  |  |  |  |  |
| 26 | I notice the smells and aromas of things. 我闻到了周围一些东西的气味或者芳香。 |  |  |  |  |  |
| 27 | Even when I’m feeling terribly upset, I can find a way to put it into words. 即使是非常不安的时候，我也能找到词语来表达它。 |  |  |  |  |  |
| 28 | I rush through activities without being really attentive to them. 我草草地做完一些事情，没有真正集中注意力。 |  |  |  |  |  |
| 29 | When I have distressing thoughts or images, I am able just to notice them without reacting. 当有烦恼情绪时，我能做到只是去注意，而不做出相应反应。 |  |  |  |  |  |
| 30 | I think some of my emotions are bad or inappropriate and I shouldn’t feel them. 我想有些情绪是不对或者不合适的，我不应该体验到他们。 |  |  |  |  |  |
| 31 | I notice visual elements in art or nature, such as colors, shapes, textures, or patterns of light and shadow. 我注意到艺术品和自然事物中一些视觉元素，如颜色形状光影。 |  |  |  |  |  |
| 32 | My natural tendency is to put my experiences into words. 我总是倾向于用词语来描述我的体验。 |  |  |  |  |  |
| 33 | When I have distressing thoughts or images, I just notice them and let them go. 当我有令人痛苦的想法或景象时，我通常只是去注意它，顺其自然。 |  |  |  |  |  |
| 34 | I do jobs or tasks automatically without being aware of what I’m doing. 我总是自动地工作或完成某项任务，而没有意识到我在做什么。 |  |  |  |  |  |
| 35 | When I have distressing thoughts or images, I judge myself as good or bad depending what the thought or image is about. 通常我有令人困扰的想法或景象时，我会根据当时脑中所想来判断自己是对是错。 |  |  |  |  |  |
| 36 | I pay attention to how my emotions affect my thoughts and behavior. 我会去注意，我的情绪是如何影响我的想法和行为的。 |  |  |  |  |  |
| 37 | I can usually describe how I feel at the moment in considerable detail. 我通常能特别详细地描述出我此刻的感觉。 |  |  |  |  |  |
| 38 | I find myself doing things without paying attention. 我发现自己做事情的时候，不专心在所做的事上。 |  |  |  |  |  |
| 39 | I disapprove of myself when I have irrational ideas. 当不理智的想法出现，我会自我否决。 |  |  |  |  |  |

(2). Can you tell us how anxious you get, if at all, with your dental visit? Please indicate by marking the appropriate box. 如果有以下情况发生，您会有对应的哪种反应？根据您的实际情况选择对应选项

| Please rate each of the following statements with the number that best describes your own opinion of what is generally true for you. | | Not anxious  =1  不焦虑 | Slightly  anxious  =2  轻微焦虑 | Fairly  anxious =3  轻度焦虑 | Very anxious =4  非常焦虑 |
| --- | --- | --- | --- | --- | --- |
| 1 | If you went to your Dentist for TREATMENT TOMORROW, how would you feel? 如果你明天要做口腔治疗，感觉如何？ |  |  |  |  |
| 2 | If you were sitting in the WAITING ROOM (waiting for treatment), how would you feel? 当你坐在候诊室里（等待治疗），感觉如何? |  |  |  |  |
| 3 | If you were about to have a TOOTH DRILLED, how would you feel? 如果你的牙齿要被钻孔，感觉如何？ |  |  |  |  |
| 4 | If you were about to have your TEETH SCALED AND POLISHED, how would you feel? 如你的牙齿要被打磨抛光，感觉如何？ |  |  |  |  |
| 5 | If you were about to have a LOCAL ANESTHETIC INJECTION in your gum, above an upper back tooth, how would you feel? 要在你的牙龈内注射麻醉（上颌后牙区），感觉如何？ |  |  |  |  |

(3). Please read the following questions carefully and choose the most suitable answer. 请仔细阅读每个题目，选择下面描述的内容与自己实际情况相符合的程度，选择相应选项。

| Please rate each of the following statements with the number that best describes your own opinion of what is generally true for you. | | Never or very rarely true =1  不符合 | Rarely true  =2  较不符合 | Sometimes true =3  不确定 | Often true =4  较符合 | Very often or always  True  =5  完全符合 |
| --- | --- | --- | --- | --- | --- | --- |
| 1 | I would rather do something that requires little thought than something that is sure to challenge my thinking abilities.  我宁愿做那些不太需要思考的事情，而不是那些一定会挑战我思考能力的事情 |  |  |  |  |  |
| 2 | I don't like to have the responsibility of handling a situation that requires a lot of thinking.  我不喜欢对需要很多思考的事情负责任 |  |  |  |  |  |
| 3 | I would prefer complex to simple problems  与简单的问题相比，我更喜欢复杂的问题 |  |  |  |  |  |
| 4 | I try to anticipate and avoid situations where there is a likely chance, I will have to think in depth about something.  我试图去预测并避免那些很可能要我深入思考的事情 |  |  |  |  |  |
| 5 | I find little satisfaction in deliberating hard and for long hours.  对某件事情长时间的思考努力给我很少的满足感 |  |  |  |  |  |
| 6 | Thinking is not my idea of fun.  思考对我来说不是件很有趣的事情 |  |  |  |  |  |
| 7 | The notion of thinking abstractly is not appealing to me.  抽象思考这件事并不能吸引我 |  |  |  |  |  |
| 8 | I prefer my life to be filled with puzzles that I must solve.  我更喜欢生活中充满我必须解决的难题 |  |  |  |  |  |
| 9 | Simply knowing the answer rather than understanding the reasons for the answer to a problem is fine with me.  知道答案而无需了解背后的原因对我来说就很不错 |  |  |  |  |  |
| 10 | I don't reason well under pressure.  我在压力状态下不能很好的推理 |  |  |  |  |  |
| 11 | The idea of relying on thought to make my way to the top does not appeal to me.  依靠思考来成为人上人的主意并不能吸引我 |  |  |  |  |  |
| 12 | I prefer to talk about international problems rather than to gossip or talk about celebrities.  我更喜欢谈论国际问题而不是八卦或谈论名人 |  |  |  |  |  |
| 13 | Learning new ways to think doesn't excite me very much.  学习新的思考方式并不能使我很兴奋 |  |  |  |  |  |
| 14 | I would prefer a task that is intellectual, difficult, and important to one that is somewhat important but does not require much thought.  相比有些重要但不需要很多思考的任务，我更喜欢需要用智力的，有些难，重要的任务 |  |  |  |  |  |
| 15 | I generally prefer to accept things as they are rather than to question them. (R)  通常我更喜欢接受事物本身而不是质疑他们 |  |  |  |  |  |
| 16 | It is enough for me that something gets the job done, I don't care how or why it works.  对我来说只要完成工作就够了，我并不在意它是如何运作的或为什么能够运作 |  |  |  |  |  |
| 17 | I tend to set goals that can be accomplished only by expending considerable mental effort.  我倾向于设立需要耗费大量脑力才能完成的目标 |  |  |  |  |  |
| 18 | I have difficulty thinking in new and unfamiliar situations.  我很难在新的和不熟悉的情况下思考 |  |  |  |  |  |
| 19 | I feel relief rather than satisfaction after completing a task that required a lot of mental effort.  在完成一项需要耗费很多脑力的任务后，我感到轻松而不是满足 |  |  |  |  |  |

(4). Your age您的年龄:____________________

(5). Your gender您的性别:

○Male男 ○Female女

(6). Your nationality您的国籍:

○China中国 ○Korea韩国 ○Indonesia 印尼 ○Other 其他
